# Once- versus twice-daily direct oral anticoagulants after ischemic stroke in atrial fibrillation – A post-hoc analysis of the ELAN trial

**DOI:** 10.1093/esj/23969873251360974

**Published:** 2026-01-01

**Authors:** Alexandros A Polymeris, Jean-Benoît Rossel, Masatoshi Koga, Daniel Strbian, Adhiyaman Vedamurthy, Manju Krishnan, Mattia Branca, Thomas Meinel, Espen Saxhaug Kristoffersen, Takeshi Yoshimoto, Kanta Tanaka, Takenobu Kunieda, Yusuke Yakushiji, Jochen Vehoff, Kosuke Matsuzono, Peter Slade, Jelle Demeestere, Alexander Salerno, Nicoletta G Caracciolo, Dimitri Hemelsoet, Stefan T Engelter, Elias Auer, Thomas Horvath, David J Seiffge, Martina Goeldlin, Jesse Dawson, Urs Fischer

**Affiliations:** Department of Neurology and Stroke Center, University Hospital Basel and University of Basel, Basel, Switzerland; Stroke Division, Department of Neurology, Beth Israel Deaconess Medical Center, Harvard Medical School, Boston, MA, USA; Department of Clinical Research, University of Bern, Bern, Switzerland; Department of Cerebrovascular Medicine, National Cerebral and Cardiovascular Center, Suita, Japan; Department of Neurology, Helsinki University Hospital, Helsinki, Finland; Glan Clwyd Hospital, Betsi Cadwaladr University Local Health Board, Rhyl, UK; Stroke Unit, Morriston Hospital, Swansea Bay University Health Board, Swansea, UK; Department of Clinical Research, University of Bern, Bern, Switzerland; Department of Neurology, Inselspital, Bern University Hospital and University of Bern, Bern, Switzerland; Department of General Practice, University of Oslo, Oslo, Norway; Department of Neurology, Akershus University Hospital, Lørenskog, Norway; Department of Neurology, National Cerebral and Cardiovascular Center, Suita, Japan; Department of Stroke and Cerebrovascular Diseases, University of Tsukuba Hospital, Tsukuba, Japan; Department of Cerebrovascular Medicine, National Cerebral and Cardiovascular Center, Suita, Japan; Department of Neurology, Kansai Medical University, Hirakata, Japan; Department of Neurology, Kansai Medical University, Hirakata, Japan; Department of Neurology, Health Ostschweiz (HOCH), Cantonal Hospital St. Gallen, St. Gallen, Switzerland; Division of Neurology, Department of Medicine, Jichi Medical University, Shimotsuke, Japan; Stroke Unit, Morriston Hospital, Swansea Bay University Health Board, Swansea, UK; KU Leuven, Department of Neurosciences, Experimental Neurology, and the Department of Neurology, University Hospitals Leuven, Leuven, Belgium; Department of Neurology, University Hospital Lausanne, University of Lausanne, Lausanne, Switzerland; Department of Human Neurosciences, University La Sapienza, Rome, Italy; Department of Neurology, Ghent University Hospital, Ghent, Belgium; Department of Neurology and Stroke Center, University Hospital Basel and University of Basel, Basel, Switzerland; Department of Rehabilitation and Neurology, University Department of Geriatric Medicine FELIX PLATTER, University of Basel, Basel, Switzerland; Department of Neurology, Inselspital, Bern University Hospital and University of Bern, Bern, Switzerland; Department of Neurology, Inselspital, Bern University Hospital and University of Bern, Bern, Switzerland; Department of Neurology, Inselspital, Bern University Hospital and University of Bern, Bern, Switzerland; Department of Neurology, Inselspital, Bern University Hospital and University of Bern, Bern, Switzerland; School of Cardiovascular and Metabolic Health, College of Medical, Veterinary & Life Sciences, Queen Elizabeth University Hospital, Glasgow, UK; Department of Neurology, Inselspital, Bern University Hospital and University of Bern, Bern, Switzerland

**Keywords:** Direct oral anticoagulants, once-daily, twice-daily, regimen, ischemic stroke, atrial fibrillation

## Abstract

**Introduction:**

Whether the risk-benefit profile of once-daily versus twice-daily direct oral anticoagulants (DOAC) differs after atrial fibrillation(AF)-associated ischemic stroke is unclear. We explored this in a post-hoc analysis of ELAN trial data (NCT03148457).

**Patients and methods:**

We compared the risk of the primary outcome (recurrent ischemic stroke, systemic embolism, intracranial hemorrhage (ICH), major extracranial bleeding, vascular death) from treatment initiation to the trial’s 90-day follow-up in participants treated with once-daily or twice-daily DOAC after AF-associated stroke using Firth’s logistic and Cox proportional hazards regression in unadjusted, inverse-probability-of-treatment-weighted and augmented-inverse-probability-weighted models to address confounding. Secondary outcomes were the primary outcome components and non-major bleeding. We calculated the net clinical benefit (NCB) of twice-daily over once-daily DOAC by subtracting the weighted rate of excess bleeding attributable to twice-daily DOAC from the rate of excess ischemic events possibly prevented by twice-daily DOAC.

**Results:**

We analyzed 1890/2013 (94%) participants (median age 77 years, 45% female), of whom 384 (20%) received once-daily and 1506 (80%) twice-daily DOAC. The primary outcome occurred in 64 (3.4%) participants, and did not differ between DOAC types in logistic (OR_unadjusted_ 0.89 (95% CI 0.50–1.66); OR_weighted_ 1.34 (0.71–2.79); OR_augmented_ 1.45 (0.81–3.21); twice-daily vs once-daily DOAC) nor in Cox models. We identified no clear differences in any secondary outcome. NCB analysis revealed a near-neutral net effect of twice-daily versus once-daily DOAC (+0.28 to +0.67 weighted events possibly prevented/100 person-months for ICH weights 1.5–3.3).

**Discussion and conclusion:**

The risk-benefit profile of once-daily versus twice-daily DOAC after AF-associated ischemic stroke does not seem to differ.

## Graphical Abstract

**Graphical abstract:**
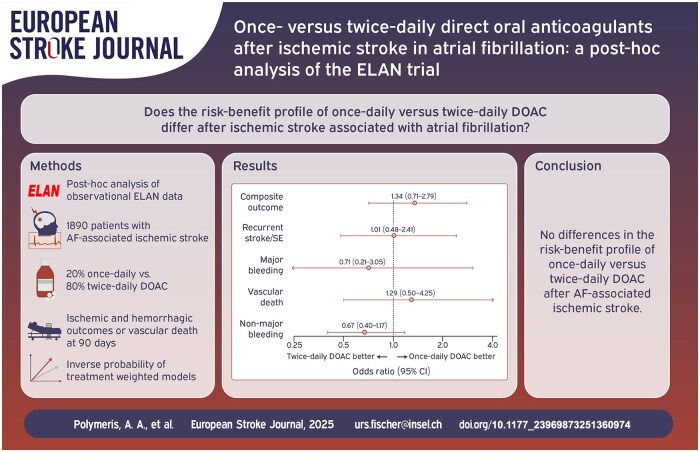

## Introduction

Direct oral anticoagulants (DOAC), comprising the factor-Xa-inhibitors apixaban, edoxaban and rivaroxaban, and the thrombin-inhibitor dabigatran, are the mainstay of treatment to prevent ischemic stroke recurrence in patients with atrial fibrillation (AF).^1–3^ While edoxaban and rivaroxaban are dosed once daily, apixaban and dabigatran follow a twice-daily dosing regimen.^3^ There are no guideline recommendations as to whether a specific DOAC type (once-daily or twice-daily) should be favored.^1–3^ To date, no large-scale, phase-3, randomized head-to-head comparisons of the risks and benefits of these DOAC types have been conducted. Several studies addressed this question in the general AF patient population by synthesizing data of direct randomized comparisons from phase-2 dose-finding trials,^4^ performing indirect comparisons in network metanalyses of phase-3 trial data,^5, 6^ or using non-randomized observational data.^7, 8^ However, results were inconsistent and data specific to patients with a recent ischemic stroke are scarce.

This knowledge gap is important, because DOAC are nowadays increasingly used in the early phase after ischemic stroke, supported by the findings of several investigations about the timing of treatment initiation after AF-associated ischemic stroke,^9^ such as the *Early versus Late Initiation of Direct Oral Anticoagulants in Post-ischemic Stroke Patients with Atrial Fibrillation* (ELAN) and other trials.^10–12^ In the post-stroke phase, the risk of recurrent stroke or other ischemic events and the risk of bleeding – particularly intracranial hemorrhage (ICH) – may not reflect those in stable AF patients without recent stroke, and both are thought to be high.^13^ Compared to once-daily DOAC, it has been suggested that twice-daily DOAC achieve more stable drug plasma levels with lower peak-to-trough variability,^14^ which might translate to both better safety and effectiveness in the high-risk post-stroke phase. Twice-daily DOAC might also be more ‘forgiving’ in the presence of non-adherence,^14^ but adherence may generally be higher with once-daily rather than twice-daily DOAC.^15^ This poses another important concern in patients with recent stroke who may have neurological and cognitive deficits interfering with their medication intakes,^16^ but no definitive data exist.

We previously investigated the risk-benefit profile of once-daily versus twice-daily DOAC in patients with recent AF-associated ischemic stroke in a single-center observational study, but identified no signal that one DOAC type might be more advantageous than the other.^17^ In this post-hoc analysis of the ELAN randomized trial, we explored this question using high-quality data from a larger, multicenter, international cohort.

## Methods

### Study design and participants

Exploratory post-hoc analysis of the ELAN international randomized controlled trial (NCT03148457) conducted across 103 sites in 15 countries in Europe, the Middle East, and Asia between November 6, 2017, and September 12, 2022. ELAN’s methodology, data collection methods, and main results are described elsewhere.^10, 18^ In brief, ELAN randomized participants with AF-associated acute ischemic stroke to early (<48 h after minor and moderate stroke, 6–7 d after major stroke) versus late (3–4 d after minor, 6–7 d after moderate, 12–14 d after major stroke) DOAC initiation. The choice of the specific DOAC type (once-daily or twice-daily) and agent (apixaban/dabigatran/edoxaban/rivaroxaban) used in each participant was at the discretion of the local treating physicians. Patients with severe renal impairment (i.e. creatinine clearance <15–30 ml/min as defined in the summary of product characteristics of the respective agent) were excluded from participation, as were those with therapeutic anticoagulation at stroke onset or with severe hemorrhagic transformation of the ischemic infarct. Stroke and treatment details, as well as risk factors and comorbidities were collected at baseline in a standardized manner, and participants were followed-up up to 90 days after randomization for the outcomes outlined below, all adjudicated centrally in a blinded fashion. All study data were gathered by local investigators and entered in a web-based database hosted by the Clinical Trial Unit (CTU) Bern, Switzerland.

In this study, we included all evaluable ELAN participants who (i) survived up to DOAC initiation and actually initiated treatment (and had available information on DOAC type and initiation time), and (ii) did not experience any study outcome before DOAC initiation. Including only participants without outcome events before DOAC initiation avoids misattribution of events to a treatment that had not yet been started. Moreover, (iii) we excluded participants with missing 90-day outcome data (due to consent withdrawal, loss to follow-up, or non-vascular death).

### Exposure and outcomes

Main exposure was the type of DOAC, that is, once-daily or twice-daily, on which study participants were started.

Primary outcome was a composite of recurrent ischemic stroke, systemic embolism (SE), symptomatic ICH, major extracranial bleeding (MEB) and vascular death from DOAC initiation up to the trial’s 90-day follow-up. The types of events comprising the composite outcome is in keeping with the main trial,^10, 18^ and their definitions were reported previously.^19, 20^ Unlike the main trial, the baseline timepoint in this study was defined as the time of DOAC initiation – rather than the earlier time of randomization to ELAN’s intervention – to avoid immortal time bias. Also in contrast to the main trial, in which 30-day events comprised the primary composite outcomes, here we chose to analyze all events up to the 90-day trial follow-up, since the longer timeframe is most relevant to this study’s aim.

Secondary outcomes were (i) major ischemic events (composite of recurrent ischemic stroke and SE), (ii) major bleeding events (composite of MEB and symptomatic ICH), (iii) vascular death, and (iv) non-major bleeding, all considered from DOAC initiation up to the trial’s 90-day follow-up and defined as described previously.^19,20^

### Statistical analysis

We present baseline characteristics according to DOAC type using descriptive statistics. We compared categorical or continuous data using the chi-square-test and the Mann–Whitney-*U*-test, respectively.

We analyzed the outcomes without any formal hypothesis testing using (i) Firths’ logistic regression (considering the small event numbers) and (ii) Cox proportional hazards regression. For both modelling approaches we used (i) simple unadjusted models including only DOAC type (once-daily or twice-daily; once-daily as reference) as the sole independent variable without further adjustments, (ii) weighted models using stabilized inverse probability of treatment weighting (SIPTW),^21^ and (iii) augmented models using augmented inverse probability weighting (AIPW) as a doubly-robust method.^22^

With the latter two methods we aimed to provide an unbiased estimate of the treatment effect by controlling for potential confounding through baseline imbalances between DOAC types. In both methods, we derived treatment weights from propensity scores estimated conditional on the following potentially outcome-modifying baseline variables, as in prior research:^23^ age; sex; stroke characteristics (National Institutes of Health Stroke Scale [NIHSS] score; infarct size (minor/moderate/major); etiology (AF-only/AF-plus);^20^ hemorrhagic transformation^24^); risk factors/comorbidities (hypertension; diabetes; dyslipidemia; smoking; history of stroke, transient ischemic attack (TIA), or SE; vascular disease (myocardial infarction or peripheral artery disease); heart failure; creatinine clearance; pre-stroke modified Rankin Scale (mRS) score⩽2; and treatment details (acute reperfusion therapy; ELAN randomization group (early/late); DOAC dose (full/reduced); antiplatelet use before DOAC initiation). Justified by the overall low missingness rate (<10% for categorical data, no missingness for continuous data), we used single imputation for missing categorical data used for weighting by sampling from a binary distribution with a probability derived from the prevalence observed in the available data. We truncated extreme weights to the 99th percentile of their distribution and assessed the balance of baseline characteristics before and after weighting using the standardized mean difference (SMD). In AIPW, the outcome model additionally included the following potential outcome modifiers, in line with previous research:^23^ NIHSS, infarct size (minor/moderate/major), ELAN randomization group (early/late), CHA_2_DS_2_-VASC score, and creatinine clearance.

For all models, we report the model-based odds or hazard ratio (OR or HR) estimates along with 95% confidence intervals (95% CI) as measure of the estimates’ precision, but refrain from presenting *p*-values or specifying significance thresholds. We estimated the weighted Cox models using robust standard errors. For the augmented models, we calculated 95% CI based on 10,000 bootstrap replications.

We also analyzed the net clinical benefit (NCB) of twice-daily versus once-daily DOAC using established methodology as in prior research,^19, 23^ with weighting of the type of events for their impact on death or disability relative to recurrent ischemic stroke. We calculated NCB by subtracting the weighted rate of excess bleeding events attributable to twice-daily DOAC from the rate of excess ischemic events possibly prevented by twice-daily DOAC, according to the following formula:



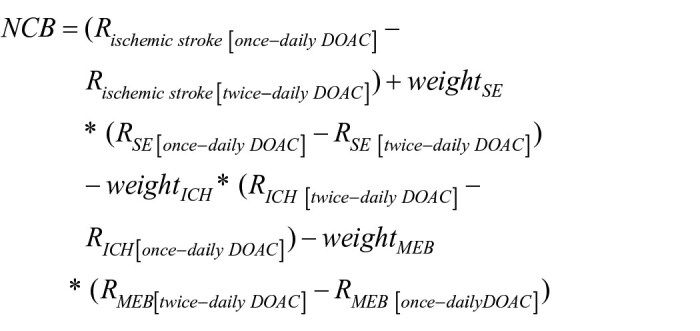



where *R* represents the incidence rate of the respective outcome and was derived from the weighted Cox model. Weight values were derived from previous empirical research,^25–28^ with ischemic stroke being assigned a weight of 1.0 (reference event), SE a weight of 0.9 (weight_SE_), and MEB a weight of 0.7 (weight_MEB_), while the weight for ICH varies from 1.5 to 3.3 (weight_ICH_). We performed the NCB analysis across the entire range of ICH weights, as in prior research.^19, 23^ We report NCB as events possibly prevented per 100 person-months along with 95%-CI, calculated based on 1,000 bootstrap replications. For the NCB we considered all participants except those with death as first outcome.

Statistical analyses were performed using Stata v.18.0 and R v.4.4.1. We conducted this study in accordance with the STROBE statement.

## Results

Of 2013 ELAN trial participants, 26 did not initiate DOAC, 29 experienced study outcomes before DOAC initiation, while 68 had missing 90-day outcome data, leaving 1890 participants (94%) available for analysis (Supplemental Figure S1).

### Baseline characteristics

Of those, 384 (20%) participants initiated once-daily DOAC and 1506 (80%) twice-daily DOAC at a median (IQR) of 3 (2–6) days after stroke onset. Compared to participants with twice-daily DOAC, those with once-daily DOAC were older, had higher NIHSS scores, their infarct size was more commonly classified as major stroke, had worse renal function and higher prevalence of previous ischemic stroke/TIA and heart failure but lower prevalence of dyslipidemia, and more commonly received reduced DOAC doses (Table 1). Weighting achieved good balance (absolute SMD < 0.05) across all baseline characteristics (Supplemental Figure S2).

**Table 1.** Baseline characteristics.

	Total (*N* = 1890)	Once-daily DOAC (*N* = 384)	Twice-daily DOAC (*N* = 1506)	p value
Age, median [IQR]	77 [70, 84]	79 [73, 86]	77 [70, 83]	<0.001
Female sex, *n/N* (%)	848/1890 (44.9%)	168/384 (43.8%)	680/1506 (45.2%)	0.62
*Stroke characteristics*				
NIHSS score (at trial randomization), median[IQR]	3 [1, 6]	4 [1, 8.5]	3 [1, 5]	<0.001
Stroke classification (based on infarct size)				0.09
Minor, *n/N* (%)	718/1890 (38.0%)	145/384 (37.8%)	573/1506 (38.0%)	
Moderate, *n/N* (%)	752/1890 (39.8%)	139/384 (36.2%)	613/1506 (40.7%)	
Major, *n/N* (%)	420/1890 (22.2%)	100/384 (26.0%)	320/1506 (21.2%)	
Atrial fibrillation-plus stroke etiology, *n/N* (%)	317/1890 (16.8%)	60/384 (15.6%)	257/1506 (17.1%)	0.50
Hemorrhagic transformation, *n/N* (%)	231/1850 (12.5%)	48/372 (12.9%)	183/1478 (12.4%)	0.79
*Risk factors and comorbidities*				
Hypertension, *n/N* (%)	1274/1872 (68.1%)	261/380 (68.7%)	1013/1492 (67.9%)	0.77
Diabetes mellitus, *n/N* (%)	314/1876 (16.7%)	59/380 (15.5%)	255/1496 (17.0%)	0.48
Dyslipidemia, *n/N* (%)	819/1837 (44.6%)	135/381 (35.4%)	684/1456 (47.0%)	<0.001
Current/past smoking, *n/N* (%)	469/1787 (26.2%)	108/365 (29.6%)	361/1422 (25.4%)	0.10
History of ischemic stroke or TIA, *n/N* (%)	316/1877 (16.8%)	79/380 (20.8%)	237/1497 (15.8%)	0.02
History of systemic embolism, *n/N* (%)	46/1874 (2.5%)	9/380 (2.4%)	37/1494 (2.5%)	0.90
History of myocardial infarction, *n/N* (%)	156/1872 (8.3%)	36/380 (9.5%)	120/1492 (8.0%)	0.37
Heart failure, *n/N* (%)	114/1761 (6.5%)	35/345 (10.1%)	79/1416 (5.6%)	0.002
Peripheral artery disease, *n/N* (%)	74/1829 (4.0%)	14/373 (3.8%)	60/1456 (4.1%)	0.75
CHA_2_DS_2_-VASc score, median [IQR]	5 [4, 6]	5 [4, 6]	5 [4, 6]	0.05
Creatinine clearance (ml/min), median [IQR]	71 [58, 86]	68 [55, 85]	72 [59, 87]	0.003
Pre-stroke mRS ⩽ 2, *n/N* (%)	1693/1888 (89.7%)	336/384 (87.5%)	1357/1504 (90.2%)	0.12
*Treatment details*				
Acute reperfusion therapy, *n/N* (%)	904/1852 (48.8%)	145/351 (41.3%)	759/1501 (50.6%)	0.002
Allocation to early DOAC initiation, *n/N* (%)	959/1890 (50.7%)	191/384 (49.7%)	768/1506 (51.0%)	0.66
Reduced DOAC dose, *n/N* (%)	350/1890 (18.5%)	108/384 (28.1%)	242/1506 (16.1%)	<0.001
Antiplatelet use before DOAC initiation, n/N (%)	1027/1890 (54.3%)	202/384 (52.6%)	825/1506 (54.8%)	0.44

### Primary outcome

**Figure 1.**
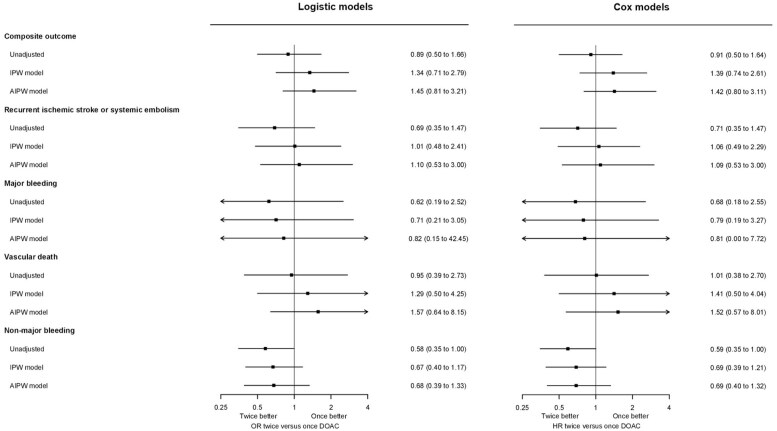
Unadjusted, inverse-probability-of-treatment-weighted, and augmented-inverse-probability-weighted odds ratio (OR) and hazard ratio (HR) estimates with 95%-CI for the effect of twice-daily versus once-daily DOAC on study outcomes.

Among 1890 participants, we observed 64 primary composite outcome events from DOAC initiation up to the trial’s 90-day follow-up, consisting of 29 recurrent ischemic strokes, 9 SE, 8 MEB, 3 symptomatic ICH, and 25 vascular deaths. Table 2 shows all outcomes according to DOAC type. In unadjusted logistic models, DOAC type was not associated with the primary outcome (OR 0.89, 95% CI 0.50–1.66 for twice-daily vs once-daily DOAC), which held true after accounting for confounding in both SIPTW and AIPW models (OR 1.34 (0.71–2.79) and 1.45 (0.81–3.21), respectively.) Cox models yielded virtually unchanged results (Figure 1).

**Table 2.** Study outcomes.

90-day outcomes	Total (*N* = 1890)	Once-daily DOAC (*N* = 384)	Twice-daily DOAC (*N* = 1506)
Primary composite outcome, *N* (%)	64 (3.4%)	14 (3.6%)	50 (3.3%)
Major ischemic event, *N* (%)	38 (2.0%)	10 (2.6%)	28 (1.9%)
Recurrent ischemic stroke, *N* (%)	29 (1.5%)	8 (2.1%)	21 (1.4%)
Systemic embolism, *N* (%)	9 (0.5%)	2 (0.5%)	7 (0.5%)
Major bleeding, *N* (%)	11 (0.6%)	3 (0.8%)	8 (0.5%)
Major extracranial bleeding, *N* (%)	8 (0.4%)	2 (0.5%)	6 (0.4%)
Symptomatic intracranial hemorrhage, *N* (%)	3 (0.2%)	1 (0.3%)	2 (0.1%)
Vascular death, *N* (%)	25 (1.3%)	5 (1.3%)	20 (1.3%)
Non-major bleeding, *N* (%)	67 (3.5%)	20 (5.2%)	47 (3.1%)

### Secondary outcomes

Upon separately analyzing the primary outcome components, DOAC type was not associated with major ischemic events, major bleeding, or vascular death, regardless of analytic approach (logistic or Cox; unadjusted, weighted or augmented models). There was a signal for a lower risk of non-major bleeding with twice-daily DOAC in unadjusted analyses, which was attenuated in the weighted and augmented models. All model-based estimates are given in Figure 1.

### NCB analysis

**Figure 2.**
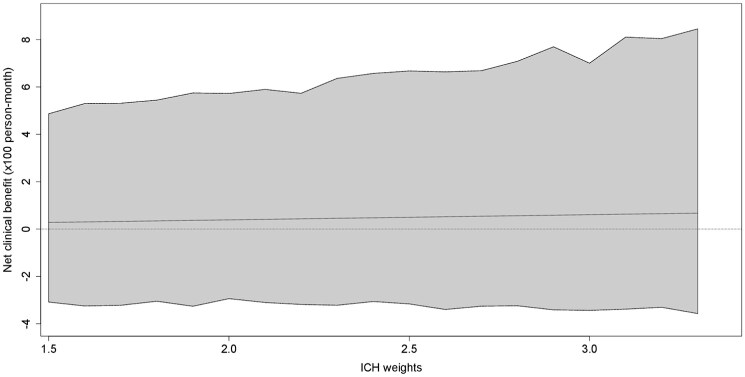
Net clinical benefit of twice-daily versus once-daily DOAC in weighted events possibly prevented per 100 person-months (solid line) with 95%-CI (grey shaded area).

The point estimates for the NCB of twice-daily versus once-daily DOAC were positive but markedly small and consistently close to 0 with wide 95% CI (+0.28 (95% CI −3.08 to +4.87) to +0.67 (−3.57 to +8.45) weighted events possibly prevented with twice-daily DOAC per 100 person-months for ICH weights 1.5 to 3.3), over the entire range of potential ICH weights (Figure 2, Supplemental Table S1).

## Discussion

This post-hoc analysis of the ELAN trial focusing on the relative risks and benefits of once-daily versus twice-daily DOAC after AF-associated ischemic stroke identified no clear differences in their effectiveness or safety. However, the generally low event numbers limit power and introduce imprecision to our estimates, precluding definitive conclusions.

The primary composite outcome of recurrent ischemic stroke, SE, symptomatic ICH, MEB and vascular death over the first 3 months after AF-associated stroke did not differ between the DOAC types regardless of the analytic approach and even after accounting for confounding through baseline imbalances. Notably, our weighting approach to constructing comparable treatment groups considered an extensive list of 19 variables from the ELAN dataset, minimizing the risk of bias through observed confounders in this well-characterized cohort. Reassuringly, doubly-robust analysis in the augmented model controlling for five potential outcome-modifying variables in addition to weighting yielded highly consistent results. In our NCB analysis, which estimates the net effect of treatment with one DOAC type versus the other by accounting for the different clinical importance of ischemic and hemorrhagic events rather than merely summing them up,^19^ we estimated a virtually neutral net effect, but with substantial imprecision.

Taken together, these findings argue against substantial differences in the overall risk-benefit profile of once-daily versus twice-daily DOAC in the early post-stroke phase, and do not support the notion that any particular DOAC type should be favored in stroke patients. These data expand on our previous study, which similarly identified no clear evidence that one type of DOAC might be more advantageous than the other, in a cohort of <1000 participants followed for an extended period of about 1.5 years after AF-associated stroke within a prospective single-center registry without formal outcome adjudication.^17^ Overcoming these limitations, the present study examined the ELAN cohort featuring almost 2000 participants in a more generalizable multicenter international setting, with an observation period focusing specifically on the early phase of 3 months post stroke, and with rigorous outcome adjudication in the context of a randomized trial. Thus, the totality of the available evidence reinforces the current guidelines, which recommend an individualized approach to the choice of DOAC type by also taking patient preferences into account.^1^

Although limited by lower event numbers, our secondary analyses examining ischemic and hemorrhagic outcomes separately were also consistent with the composite outcome analysis, revealing no signal for a differential effect of DOAC type on different types of major outcomes, including major bleeding. This is important, because safety of anticoagulation – particularly with regards to ICH – is a major concern in the early post-stroke phase.^13^ However, we did identify a weak signal for a potentially lower risk of non-major bleeding with twice-daily DOAC. This adds to previous controversy about the effects of DOAC type on non-major bleeding, with some studies identifying differences in favor of twice-daily DOAC,^7^ and others reporting no differences.^8^ Considering that non-major bleeding may be more impactful as previously thought,^29^ and that large observational studies on the comparative safety of specific DOAC agents in the general AF patient population have repeatedly shown lower bleeding risk with apixaban and dabigatran,^30–32^ both twice-daily DOAC, these findings warrant more scrutiny in future research.

Our study has the following strengths: (i) We used high-quality, rigorously collected, prospective data from a large multicenter international randomized trial with central event adjudication. (ii) We employed several lines of statistical inquiry, all leading to consistent results. We acknowledge the following limitations: (i) As a post-hoc analysis of non-randomized, unbalanced comparison groups, our study is exploratory and precludes causal inference. Despite best efforts to account for confounding using SIPTW and AIPW, residual bias through unmeasured confounders might have still influenced our findings. In particular, we did not account for medication adherence, which is important in this context but was not available for analysis. (iii) Our choice to exclude participants who died or suffered outcomes before DOAC initiation may introduce selection (survivor) bias, but is appropriate to address the most clinically relevant question about treatment effects in patients who start treatment after a stroke. (iii) The lack of differences in our study may be attributable to low power due to low numbers of events, which introduces imprecision to our estimates and prevents definitive conclusions. To demonstrate a difference in the primary outcome with confidence given our AIPW logistic model estimate, a sample of <10,000 participants would be required (Supplemental Figure S3). The individual participant data meta-analysis of ELAN and similar trials (CATALYST) might provide an opportunity for this.^33^

In conclusion, we found no clear differences in the risk-benefit profile of once-daily versus twice-daily DOAC after AF-associated ischemic stroke in the large multicenter international cohort of the ELAN trial. While these data do not support the preferential use of any particular type of DOAC in the post-stroke phase, power may have been low to detect subtler differences and investigations in even larger cohorts may be warranted.

## Supplementary Material

sj-docx-1-eso_23969873251360974

## Data Availability

The data that support the findings of this study may be made available on reasonable request to the corresponding author and after approval by the ELAN steering committee.

## ORCID iDs

Alexandros A Polymeris   https://orcid.org/0000-0002-9475-2208

Jean-Benoît Rossel   https://orcid.org/0000-0001-5803-3233

Daniel Strbian   https://orcid.org/0000-0001-9095-2344

Thomas Meinel   https://orcid.org/0000-0002-0647-9273

Espen Saxhaug Kristoffersen   https://orcid.org/0000-0002-8999-5424

Takeshi Yoshimoto   https://orcid.org/0000-0003-3178-8171

Kanta Tanaka   https://orcid.org/0000-0002-3291-5083

Jelle Demeestere   https://orcid.org/0000-0001-8186-0237

Alexander Salerno   https://orcid.org/0000-0001-8494-5527

Stefan T Engelter   https://orcid.org/0000-0003-3855-6234

David J Seiffge   https://orcid.org/0000-0003-3890-3849

Martina Goeldlin   https://orcid.org/0000-0001-5800-116X

Jesse Dawson   https://orcid.org/0000-0001-7532-2475

Urs Fischer   https://orcid.org/0000-0003-0521-4051
